# Discovery of Quinacrine as a Potent Topo II and Hsp90 Dual-Target Inhibitor, Repurposing for Cancer Therapy

**DOI:** 10.3390/molecules27175561

**Published:** 2022-08-29

**Authors:** Xin Pan, Teng-yu Mao, Yan-wen Mai, Cheng-cheng Liang, Wei-hao Huang, Yong Rao, Zhi-shu Huang, Shi-liang Huang

**Affiliations:** 1 School of Pharmaceutical Sciences, Sun Yat-sen University, Guangzhou 510006, China; 2 Guangdong Provincial Key Laboratory of New Drug Design and Evaluation, Guangzhou 510120, China

**Keywords:** HSP90 inhibitor, dual−target inhibitor, topo II inhibitor, quinacrine, the N−ATPase domains

## Abstract

Topo II and Hsp90 are promising targets. In this study, we first verified the structural similarities between Topo IIα ATPase and Hsp90α N−ATPase. Subsequently, 720 compounds from the Food and Drug Administration (FDA) drug library and kinase library were screened using the malachite green phosphate combination with the Topo II-mediated DNA relaxation and MTT assays. Subsequently, the antimalarial drug quinacrine was found to be a potential dual−target inhibitor of Topo II and Hsp90. Mechanistic studies showed that quinacrine could specifically bind to the Topo IIα ATPase domain and inhibit the activity of Topo IIα ATPase without impacting DNA cleavage. Furthermore, our study revealed that quinacrine could bind Hsp90 N−ATPase and inhibit Hsp90 activity. Significantly, quinacrine has broad antiproliferation activity and remains sensitive to the multidrug−resistant cell line MCF−7/ADR and the atypical drug−resistant tumor cell line HL−60/MX2. Our study identified quinacrine as a potential dual−target inhibitor of Topo II and Hsp90, depending on the ATP−binding domain, positioning it as a hit compound for further structural modification.

## 1. Introduction

With the development of molecular oncology, a large number of molecular targets for therapeutic intervention have been found by researching biochemical and molecular differences between normal cells and cancer cells. Both Topo II and Hsp90 are important targets for cancer chemotherapy.

Topo II plays a critical role in the process of transcription, replication, and chromosome segregation [1,2,3]. Topo II inhibitors can be divided into Topo II poisons and Topo II catalytic inhibitors [4,5]. Topo II poisons can stabilize the cleavage complex, which can be divided into interface binding or covalent binding [6]. Stabilization of the DNA cleavage complex results in the formation of permanent double−strand breaks [7,8]. For example, etoposide, doxorubicin, mitoxantrone, and idarubicin are Topo II poisons. Although they have good antitumor effects, consistent use will lead to secondary leukemia, cardiotoxicity, neurotoxicity, severe bone marrow suppression, gastrointestinal reactions, liver and kidney damage, and other side effects [9,10]. In contrast, Topo II catalytic inhibitors prevent the formation of the DNA cleavage complex [11]. Compared with Topo II poisons, Topo II catalytic inhibitors have higher efficiency and lower toxicity, so they have become a trend in the development of Topo II inhibitors. Topo II catalytic inhibitors acting on the ATPase region have been a research hotspot in recent years [12,13].

Hsp90 is another attractive target for cancer treatment. Hsp90 is upregulated in numerous tumor cells [14]. Hsp90 has been reported to be required for the maturation, activation, and stabilization of more than 200 client proteins and is essential for many cell signaling pathways [15,16]. There are four isoforms of human Hsp90, Hsp90α, Hsp90β, Grp94, and TRAP1 [17], and inducible Hsp90α is the main isoform. Hsp90 inhibitors can be classified into three categories by their different sites of action: N−terminal inhibitors, C−terminal inhibitors, and other inhibitors. Presently, several Hsp90 N−terminal inhibitors are undergoing clinical trials for the treatment of various forms of cancer [18]. However, the disadvantages of these inhibitors, such as concomitant Hsp70 induction and resistance, have hindered their further clinical applications [19].

Topo II and Hsp90 have different biological functions in vivo. However, both exhibit sustained and highly induced expressions in tumor cells, so both are important antitumor targets. However, current clinical treatment using Topo II or Hsp90 inhibitors alone is not sufficiently ideal, and combined drug research shows that Topo II poisons and Hsp90 inhibitors have a synergistic effect in tumor treatment [20,21,22,23,24]. There is a certain correlation between Topo II and Hsp90 in tumor treatment. When Topo II and Hsp90 are inhibited simultaneously, the effect of tumor treatment can be improved. However, it is still difficult to avoid drug resistance and genotoxicity caused by the DNA cleavage complex produced by Topo II poisons. By studying the crystal structure of Topo II and Hsp90, the N−terminal ATPase region of both belongs to the GHKL (Gyrase B, Hsp90, Histidine Kinase, MutL) family protein. Even though the degree of homology between these two ATP−binding domains is low (16%), ATP−binding, ATP hydrolysis, and ATP/ADP nucleotide exchange are essential for their function [23,25]. By verifying the similarity of the ATPase regions of both, here we expected to screen out dual inhibitors that could simultaneously target the N−ATPase domains of Topo II and Hsp90. In terms of mechanism, this type of dual inhibitor can inhibit Topo II and Hsp90 without generating a DNA cleavage complex and can reduce the occurrence of toxicity and side effects while improving safety and efficacy. Some researchers have reported the discovery of dual inhibitors of these two targets and a pharmacophore model for dual Topo II/Hsp90 inhibitors [26,27,28].

Drug repurposing is an excellent option for the discovery of already used drugs, lowering the cost of production, and shortening the period of delivery [29]. For example, many approved drugs have shown significant results against COVID−19 [30]. Otsuka’s team found a new target for S−trityl−L−cysteine−derived compounds distinct from their well−known mitotic kinesin Eg5 inhibition [31]. In our study, 720 compounds in the FDA drug and kinase compound libraries were screened through the malachite green phosphate assay combined with the Topo II−mediated DNA relaxation and MTT assays. Then we found that the antimalarial drug quinacrine (QA) could be a potential dual−target inhibitor of Topo II and Hsp90 depending on the ATP−binding domain. QA has been reported to have multiple indications, not only antimalaria, such as refractory giardia disease [32], and cutaneous lupus erythematosus [33]. Our study first shows that it has great potential as a dual−target inhibitor for the treatment of cancer and merits further study in the future.

## 2. Results

### 2.1. The Structural Alignment on Topo IIα and Hsp90α

To verify the similarity of the ATPase regions of Topo IIα and Hsp90α, we superimposed the crystal structures with ANP (adenylyl−imidodiphosphate) (PDB: 1ZXM, 3T1K) [34,35]. As shown in Supplementary Materials Figure S1A,B, there were some similarities in the overlap of the two ANP active domains. The β−fold at the front end of the pocket mostly overlapped, and the center of the ATPase region was positioned close to Mg^2+^. The orientation and configuration of the substrate ANP at the active site were also basically the same. As shown in Figure S1C,D, the key amino acids Asp93 of Hsp90 and Asn120 of Topo II, Asn51 of Hsp90 and Asn91 of Topo II almost overlap. Some studies have shown that these two classes combined have synergistic effects [20,21]. However, we also noticed that there was a larger active pocket area and a larger solvent exposure area in Hsp90 N−ATPase than that of Topo II, which might lead to the difficult design of this type of double target inhibitor.

### 2.2. Preliminary Screening of Topo IIα Inhibitors

Many methods have been reported for screening Topo II or Hsp90 ATPase inhibitors [36,37,38,39,40,41]. Malachite green phosphate assays have been widely reported for the detection of ATPase activity due to their simple operation and the wide availability of reagents [42,43,44,45,46]. Because most kinase inhibitors act on ATP−binding sites and are convenient for subsequent studies, we selected a commercial 80 kinase inhibitor library and 640 FDA drug library for the screening of dual Topo II and Hsp90 N−ATPase inhibitors. From the screening results of the kinase library (as shown in Figure S2A), K−B6 (staurosporine), K−F9 (palmitoyl−DL−carnitine Cl), and K−F12 (daidzein) had better inhibitory activity against Topo II ATPase than the positive drug 1,4−NQ. Among them, 15 kinase inhibitors had a relative inhibition rate of Topo II ATPase greater than 60%. Information on these kinase inhibitors is shown in Table 1, and the chemical structure is shown in Figure S3. In addition, we evaluated the relative inhibitory activity of Topo II ATPase against the existing drugs in 640 FDA drug libraries. As shown in Figure S2B–I, the relative inhibitory activity of the 20 compounds was above 40%. Information on these drugs is shown in Table 1 and the chemical structures are shown in Figure S4.

Since both Topo II and Hsp90 inhibitors had antitumor activity, an MTT assay was used to narrow the screening results. HL−60 human leukemia cell is sensitive to Topo II inhibitors, which are commonly used to evaluate the inhibitory effect of Topo II inhibitors on proliferation. As shown in Table 1, the kinase inhibitors K−B6, K−C10, K−D7, K−D9, and K−F12 showed good inhibition of HL−60 cells (IC_50_ < 10 μM), and the kinase inhibitors K−C11, K−D1, K−D6, K−D10, and K−D11 demonstrated moderate cytotoxicity (IC_50_ < 25 μM). The 2−G7, 3−H6, 4−C3, and 7−F2 (quinacrine, QA) in the FDA drug library showed good activity in inhibiting the proliferation of HL−60 cells (IC_50_ < 10 μM), and 2−B8, 2−H10, 3−D11, 5−E11, and 6−B10 had moderate cytotoxicity (IC_50_ < 25 μM).

### 2.3. QA May Be a Potential Dual−Effect Inhibitor Based on Further Screening

**Figure 1:**
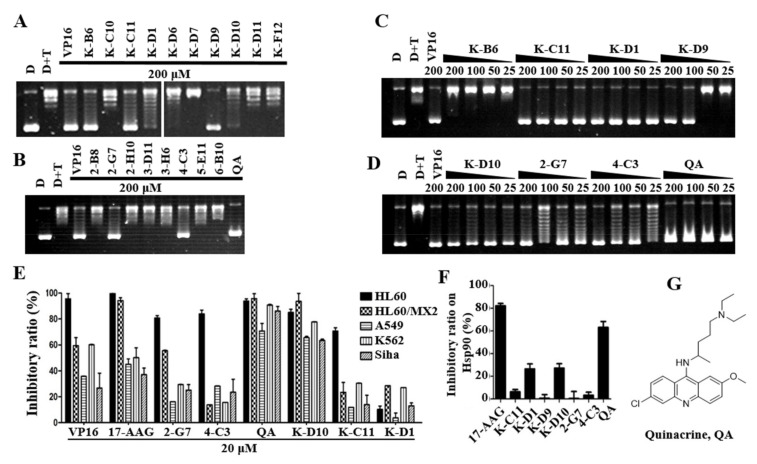
QA is a potential dual−effect inhibitor based on further screening. (**A**) Effect of kinase inhibitors on the DNA relaxation assay. (**B**) Effect of FDA−approved drugs on the DNA relaxation assay. The concentration of compounds was 200 μM (**A**,**B**). (**C**,**D**) Effect of compounds at different concentrations (200, 100, 50, and 25 μM) on the DNA relaxation assay. The concentration of VP−16 as the control was 200 μM (**A**–**D**). (**E**) Inhibitory effects of compounds at a concentration of 20 μM on HL−60, HL−60/MX2, A549, K562, and Siha cells by the MTT assay. (**F**) Inhibitory effect of compounds on Hsp90α. The Hsp90α inhibitory activity of the compounds at 100 μM was tested by the malachite green phosphate assay with 2.5 μg Hsp90α. After 3 h, the reaction was stopped, and the OD620 value was measured. (**G**) The structure of 7−F2 (quinacrine, QA).

To further evaluate the inhibitory activity of Topo II and cytotoxicity at the cell level, a Topo IIα−mediated pBR322 relaxation assay was conducted. VP−16 is a positive control that acts as a Topo II inhibitor and can inhibit DNA relaxation at a concentration of 100 μM [47]. The kinase inhibitors K−B6, K−C11, K−D1, K−D9, and K−D10, and the drugs 2−G7, 4−C3, and QA completely inhibited DNA relaxation at a concentration of 200 μM, the kinase inhibitors K−D1 and K−D9 at this concentration mostly inhibited DNA relaxation, and the remaining compounds showed very weak or no inhibitory activity (Figure 1A,B). To further evaluate the inhibitory activity of these eight compounds, we detected their inhibition in a gradient concentration. We further evaluated the abilities of these eight compounds to inhibit the activity of Topo II using a gradient concentration. As shown in Figure 1C,D, all seven compounds, except K−B6, showed good inhibitory activity. K−C11, K−D1, and QA could completely inhibit DNA relaxation at 25 μM. The results between the malachite green phosphate assay and the Topo II DNA relaxation assay were not quite consistent, which was speculated to be due to the involvement of multiple complex structural allosteric processes in the catalytic cycle formed in the Topo II DNA relaxation assay. Thus, more experiments are needed to verify and exclude false−positive results.

The seven compounds obtained from the above screening were used to test the inhibitory activity against Hsp90 by the malachite green phosphate assay. As shown in Figure 1F, QA inhibited Hsp90 at about 70% at 100 uM, which is comparable with the positive control 17AAG (83% inhibition at 100 μM). A high concentration of compounds was used here because the assay used a high concentration of the enzyme, which correlated with the inhibitory activity [48]. Here, the inhibitory activity of 17AAG is similar to the literature report [43]. The structure of QA is shown in Figure 1G.

More tumor cell lines were used for the anti−proliferation activity validation of compounds at 20 μM. As shown in Figure 1E, QA and K−D10 have good anti−proliferative activities against four tumor cell lines, and the inhibition rate of QA is above 70%. Moreover, 2−G7 and 4−C3 are more sensitive to HL−60 cells but have poor anti−proliferative activities against other tumor cell lines. K−D1 has poor anti−tumor activity (the inhibition rate is below 40%). Combining the series of screening and activity validation experiments described above, QA may be a potential dual−effect inhibitor that can simultaneously inhibit the activity of Topo IIα and Hsp90α, which was selected as a hit for further mechanistic studies.

### 2.4. QA Is a Topo II Catalytic Inhibitor with Weak DNA Intercalation

**Figure 2:**
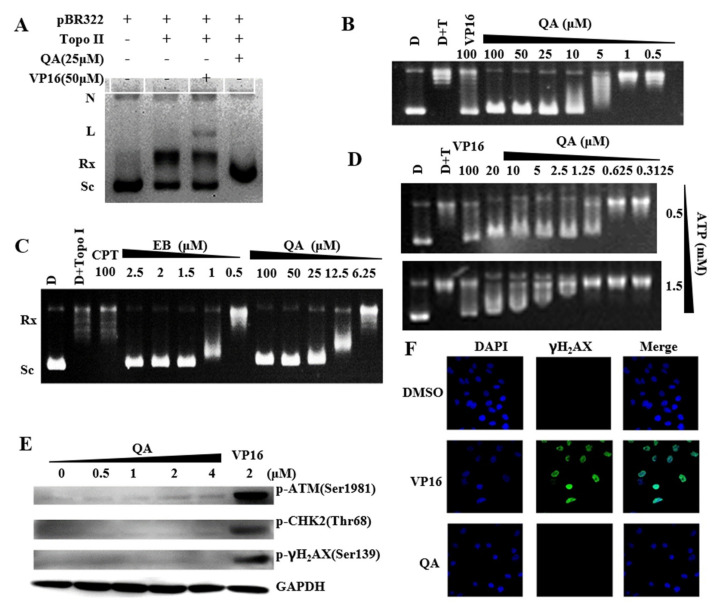
QA inhibits the activity of Topo IIα without impacting DNA cleavage. (**A**) Effects of QA on Topo II−DNA cleavage complex formation. N: nicked DNA; L: linear DNA; Rx: relaxed DNA; Sc: supercoiled DNA. (**B**) Inhibitory effect of QA on DNA relaxation by Topo II at the indicated concentration. (**C**) DNA unwinding effects of QA. (**D**) Effects of ATP concentration on Topo II inhibition of QA. The Topo IIα−mediated pBR322 relaxation assay was performed with QA (20, 10, 5, 2.5, 1.25, 0.625, 0.3125 μM) and ATP (0.5, 1.5 mM). (**E**) Expression of DNA damage pathway−related proteins with QA treatment. A549 cells were treated with QA (0.5, 1, 2, 4 μM) for 24 h. Protein levels were analyzed by western blotting. VP−16 (2 μM, Topo II poison) was used as a positive control. (**F**) Effects on γH_2_AX phosphorylation levels with QA treatment. Immunofluorescent staining of γH_2_AX in A549 cells treated with VP−16 (10 μM) or QA (2 μM) for 2 h. Cells were incubated with phospho−γH_2_AX antibody followed by incubation with anti−rabbit Alexa 488−conjugated antibody (green). Nuclei were stained with DAPI (blue). Images were captured using a fluorescence microscope.

Topo IIα generates transient double−stranded DNA breaks as part of its catalytic cycle to regulate DNA topology [49,50]. A Topo II−mediated DNA cleavage assay was used to investigate whether QA was a Topo II poison or a catalytic−type inhibitor based on the formation of nick or linear DNA. As shown in Figure 2A, VP16 (Etoposide) was a topoisomerase II poison and resulted in the substantially increased formation of linear DNA. QA could completely inhibit DNA relaxation at 10 μM (Figure 2B). The IC_50_ of QA was 6.18 μM but it could not form linear DNA and nicked DNA at 25 μM, so the mechanism of action of QA was different from that of Topo II poison VP16 and did not raise Topo II−DNA cleavage complexes. Moreover, the intercalation intensity (Figure 2C) of QA at 25 μM in the unwinding assay was similar to that of EB at 1.5 μM and was much higher than that of complete inhibition in the Topo IIα−mediated pBR322 relaxation assay. The inhibitory effect of QA on Topo II at this concentration was not mediated through the action of intercalating DNA. Furthermore, as shown in Figure 2D, with the increase in ATP concentration in the system (from 0.5 mM to 1.5 mM), the inhibitory activity of QA on Topo II decreased. The inhibitory activity of QA was related to the concentration of ATP, and both might compete for the same binding site.

In addition, western blot and immunofluorescence assays were used to detect DNA damage−induced expression of the identified marker proteins in cells after drug treatment. Significant upregulations of the marker proteins p−ATM, p−Chk2, and γH_2_AX were observed after A549 cells were treated with VP16 for 24 h, while QA treatment did not induce an increase in these proteins (Figure 2E). This result indicated that QA did not cause DNA damage in tumor cells. Similarly, immunofluorescence assays also showed that QA could not induce or increase the number of phospho−γH_2_AX foci (Figure 2F). These findings were in agreement with the results obtained for the cleavage assay, which indicated that QA might not be a Topo II poison but a catalytic inhibitor with weak intercalation of DNA.

### 2.5. QA Can Bind the N−Terminal ATP−Binding Site of Hsp90 and Inhibit Hsp90 Activity

**Figure 3:**
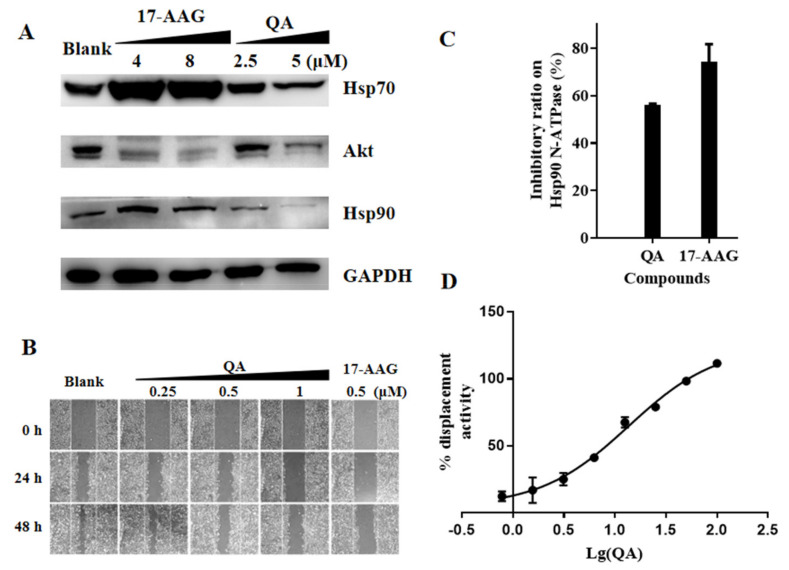
QA can bind to the ATP−binding site of Hsp90 and inhibit Hsp90 activity. (**A**) The expression level of proteins with QA treatment. A549 cells were treated with QA (2.5 and 5 μM) for 48 h. Protein levels were analyzed by western blotting. The 17−AAG (4 and 8 μM) was used as a positive control. (**B**) Effect of QA on A549 cell migration. Scratched A549 cells were treated with QA (0.25, 0.5, 1 μM) for 0, 24, and 48 h; 17−AAG (0.5 μM) was used as a positive control. (**C**) Inhibitory effect of compounds on Hsp90α N−ATPase. Hsp90α N−ATPase activity of the compounds at 100 μM was tested by the malachite green phosphate assay with 2.5 μg Hsp90α N−ATPase. After 3 h, the reaction was stopped, and the OD620 value was measured. (**D**) A competitive binding assay employing FITC−labeled geldanamycin and Hsp90α N−ATPase was performed. We used 40 nM GM−FITC, 80 nM Hsp90 N−ATPase, and QA at the indicated concentrations (100, 50, 25, 12.5, 6.25, 3.12, and 1.56 μM).

The main function of Hsp90 is to act as a molecular chaperone to facilitate the folding of many Hsp90 client proteins [15,16]. Hsp90 inhibition usually induces the degradation of its client proteins through the proteasome pathway [51]. As shown in Figure 3A, after A549 cells were treated with different concentrations of QA for 48 h, the expression of Akt was significantly downregulated at 2.5 and 5 μM. The results indicated that QA could affect the expression of Hsp90 client protein.

In general, Hsp90 N−ATPase inhibitors, such as 17−AAG, have low efficacy, likely due to the protective effects of the heat shock response induced in treated cells. As a result, resistance to Hsp90 inhibitors can occur. As shown in Figure 3A, when the Hsp90 inhibitor 17−AAG was used at concentrations of 4 and 8 μM, the expressions of Hsp90 and Hsp70 were significantly upregulated. Conversely, obvious downregulation effects on Hsp70 and Hsp90 expressions were detected following treatment with increasing concentrations of QA from 2.5–5 μM, which indicated that QA was more beneficial to avoid drug resistance compared with classical Hsp90 inhibitors.

A wound−healing assay with A549 cells was used to evaluate the effect of QA on tumor cell migration. As shown in Figure 3B, QA inhibited A549 cell migration in a dose−dependent manner. The inhibitory effect at a 1 μM concentration was comparable to that of 0.5 μM of the positive drug, 17−AAG.

In the preliminary screening experiment, QA inhibited the activity of Hsp90 to hydrolyze ATP in the malachite green phosphate assay. As shown in Figure 3C, the same result was observed when using recombinant Hsp90 N−ATPase instead of Hsp90. Next, in a validated commercial FP competitive binding assay, as shown in Figure 3D, QA displayed significant competitive displacement activity (IC_50_ = 13.63 μM) against GM−FITC. Based on the results of our FP and ATPase enzymatic activity assays, QA was identified as an Hsp90 inhibitor that bound to the ATP−/ADP−binding site in the N−terminus of Hsp90 without a feedback HSR effect.

### 2.6. Binding Model of QA on Topo II and Hsp90

**Figure 4:**
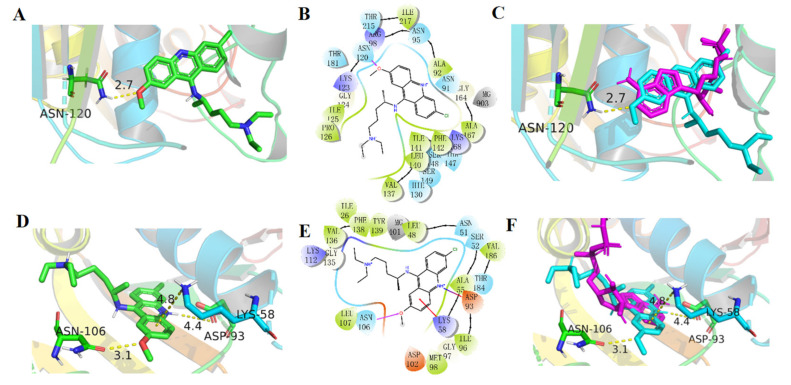
Binding model of QA on Topo II and Hsp90. (**A**,**B**) Binding model of QA on Topo II (PDB ID:1ZXM). (**C**) Overlay of QA and ANP on Topo II. QA and ANP are shown in cyan and magenta, respectively. (**D**,**E**) Binding model of QA on Hsp90 (PDB ID:3T1K). (**F**) Overlay of QA and ANP on Hsp90. QA and ANP are shown in cyan and magenta, respectively.

To further elaborate the interaction mode of QA and ATPase, molecular docking analysis was performed using Glide of Schrodinger software. In the binding model of QA on Topo II (PDB ID:1ZXM), as shown in Figure 4A–C, QA could enter the ATP−binding region of Topo II and bind to the same position as ATP. The aromatic planar structure of QA and the hydrophobic pocket of ATPase (Ile141, Val137, Phe142, Pro126, Ile125, Ala92) had hydrophobic effects. In addition, the critical residue Asn120 could form a hydrogen bond with -OCH_3_. Although the side chains of QA and ANP pointed in different directions, the aromatic planar structures of both occupy the same position and provide the strongest binding force. The docking results further confirmed that QA had a good binding capacity with Topo II ATPase.

For Hsp90, the docking results were similar to the former findings. The differences mainly reflected the pocket size, aromatic planar structure position, and binding force. As shown in Figure 4D–F, cation–π interactions formed between the critical residue Lys58 and the planar aromatic ring. The Asn106 could form a hydrogen bond with -OCH_3_ and the Asp93 could form an ionic bond with NH^+^ of the aromatic ring. Overall, in both Topo and Hsp proteins, the side chains of QA were in the external solvent−exposed region of the active site, and the aromatic ring of QA had a similar binding pattern and a hydrophobic environment, which provided the possibility of designing dual inhibitors of the targets.

### 2.7. QA Has Broad Antitumor Activity and Induces Cell Apoptosis

**Figure 5:**
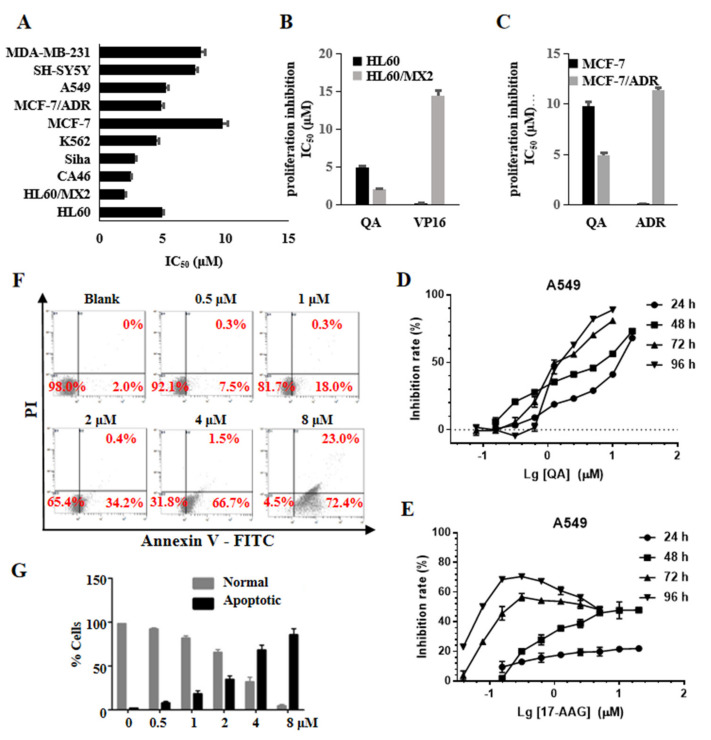
QA has broad antitumor activity and induces cell apoptosis. (**A**) IC_50_ values of QA against cancer cells. Cancer cells were treated with the indicated concentration of QA for 48 h. Cell proliferation was determined by the MTT assay. (**B**,**C**) IC_50_ comparison of QA, VP−16, and ADR against parental and resistant tumor cells. (**D**) Cell growth inhibition curves of QA against A549 at 24, 48, 72, and 96 h. (**E**) Cell growth inhibition curves of 17−AAG against A549 at 24, 48, 72, and 96 h. (**F**) Effect of QA on the apoptosis of HL−60 cells. HL−60 cells were treated with QA (0.5, 1, 2, 4, 8 μM) for 48 h, and apoptotic cell death was determined by flow cytometry. Numbers refer to the percentage of cells undergoing apoptosis. (**G**) Quantitative histogram of the data is shown in Figure 5F.

The MTT assay was used to further investigate the antiproliferative activity of QA toward a panel of tumor cell lines. As shown in Figure 5A, QA had broad antiproliferation activity, and the IC_50_ values were below 10 μM. QA remained sensitive to the multidrug−resistant cell line MCF−7/ADR and the atypical drug−resistant tumor cell line HL−60/MX2. In particular, the HL−60/MX2 cell line was generally resistant to Topo II poisons and sensitive to Topo II catalytic inhibitors. As shown in Figure 5B,C, the resistance factors (RFs) of QA on the two cell lines were 0.50 (MCF−7/ADR to MCF−7) and 0.41 (HL−60/MX2 to HL−60), which were much lower than those of control anticancer drugs, including doxorubicin (RF = 88) and VP16 (RF = 144). QA effectively inhibited the proliferation of A549 cells in a concentration and time−dependent manner (Figure 5D). Although the Hsp90 inhibitor 17−AAG has nM cytotoxicity activity (IC_50_ = 230 nM at 72 h and 75 nM at 96 h) (Figure 5E), A549 cells have a survival rate of about 30–40% at high doses (up to 5 μM) and for a long time, and even if the dose reaches 20 μM, 17−AAG cannot achieve a 50% inhibition rate at 24 and 48 h MTT assays. Combined with the results obtained from the above WB experiments (Figure 3A), this may be related to the time required for degradation of the client protein after Hsp90 is inhibited and the feedback at high doses induces a heat shock response to improve the expression of Hsps to protect tumor cells.

Annexin V−FITC/PI was used to identify apoptotic and necrotic cells by flow cytometry. As shown in Figure 5F,G, the concentration of QA increased from 0.5 to 8 μM, and the rate of apoptosis also gradually increased from 7.5% to 72.4%. These results showed that QA could effectively induce apoptosis of HL−60 cells in a concentration−dependent manner.

## 3. Discussion

Topo II and Hsp90 are important targets for cancer treatment. The inhibition of Topo II or Hsp90 can inhibit tumor growth [13,15,52]. However, current clinical therapy for Topo II or Hsp90 alone is not ideal, and combined drug research shows that Topo II poisons and Hsp90 inhibitors have a synergistic effect in tumor treatment [20,21,22]. In addition, there are structural and key amino acid similarities in the N−terminal ATPase region of Topo IIα and Hsp90α. Thus, we expect to screen out dual inhibitors that can simultaneously target Topo II and Hsp90 ATPase.

In our study, 720 compounds from the FDA drug library and kinase compound library were screened using the malachite green phosphate assay of Topo II ATPase and Hsp90 and the Topo II−mediated DNA relaxation assay, combined with the MTT assay. From the results of the preliminary screening, the kinase library (19%) showed a higher probability of screening out the Topo II and Hsp90 dual−target inhibitors than the FDA library (4%). The result may have been due to the ability of the kinase, Topo II, and Hsp90 to interact with the same ligand ATP, and a certain degree of similarity in the mode of action. However, the absence of good Topo II and Hsp90 dual−target hits in the kinase library suggested that the kinase family was vastly different from Hsp90 and Topo II, especially in terms of function. Interestingly, more than half of the compounds that were screened out in the malachite green phosphate assay of Topo II ATPase were PKC kinase inhibitors, suggesting structural similarities between them.

The antimalarial drug QA (7−F2) has been found to have good antitumor activity in vitro and in vivo, and its antitumor mechanism involves multiple targets and pathways, including heat shock response (HSR) and Topo II inhibition [53,54,55,56]. However, there is no data in the existing literature to support whether QA is a poison or a catalytic inhibitor of Topo II; moreover, there is no data to support QA as a class of the Hsp90 inhibitor. Here, we demonstrated that QA could inhibit the activity of Topo II and Hsp90 and bind to the N−ATPase domains of both. Unlike conventional Topo II inhibitors, such as VP16, QA does not produce broken DNA at either the enzymatic or cellular level, and it is a Topo II catalytic inhibitor, unlike traditional Hsp90 inhibitors, such as 17−AAG, which is shown to induce HSR to improve the expression of Hsps and improve the survival rate of tumor cells in incubation for long periods of time and under high concentrations (Figure 5D,E). QA downregulates the expression of chaperone and client protein and does not produce feedback to induce HSR (Figure 3A). These results also suggest that the dual inhibitors of Hsp90 and Topo II have a greater advantage than the two targets being suppressed alone. This feature is beneficial to help solve the current challenges encountered in the clinical application of HSP90 inhibitors.

Competitive experiments using GM−FITC as a probe confirmed that QA was bound to the ATP−binding site of HSP90 (Figure 3D). However, we did not confirm whether QA could bind to the ATP−binding site of Topo II due to the lack of a corresponding probe. This binding was partially illustrated in the ATP competition assay but it was not clear enough due to the complicating configuration changes with the dynamic ATP depletion of Topo II. The binding mode was simulated by molecular docking (Figure 4). Although the sizes of the ATP−binding sites for Topo II and Hsp90 vary, a key hydrogen bond was formed between QA and Asn120 of Topo II and Asn106 of Hsp90. The side chain and aromatic ring of QA have a similar binding pattern and hydrophobic environment. These findings also provide very important structural information for the next step of structural modification.

In summary, our study provides a hit compound QA that can target the ATP−binding domain of Topo II and Hsp90 with significant antiproliferation and remains sensitive to resistant cell lines. QA has the potential to overcome the shortcomings of Topo II and Hsp90 alone. These results imply that QA acts as a novel dual inhibitor of Topo II and Hsp90 with promising clinical applications. Structural optimization efforts are ongoing based on structural information about the action of QA with Topo II and Hsp90.

## 4. Materials and Methods

### 4.1. Reagents and Antibodies

A total of 720 compounds were purchased from the National Compound Resource Center, including 80 kinase inhibitors and 640 FDA−approved drug libraries. The positive control drugs etoposide (VP−16, Selleck, s1225), 17−AAG (Selleck, s1141), and quinacrine 2HCl (Selleck, s4255) were purchased as standards. All compounds were >95% pure by HPLC analysis. Geldanamycin−FITC (Abcam, ab141589), Topo IIα relaxation assay kit (Inspiralis, HTR202, UK), pBR322 DNA (Takara, 3050), topoisomerase I (Takara, 2240 A), and HSP90α (Abcam, ab80369) were purchased for the inhibitory assay. Antibodies phospho−ATM (Ser1981, no. 5883), phospho−Chk2 (Thr68, no. 2197), phospho−γH_2_AX (Ser139, no. 9718), GAPDH (no. 5174), HSP90α (no. 8165), HSP70 (no. 4873), AKT (no. 4691), anti−rabbit IgG−HRP, and SignalFire™ elite ECL reagent (no. 12757) were obtained from Cell Signaling Technology. Annexin V−FITC and propidium iodide were obtained from KeyGEN BioTECH; 4′,6−diamidino−2−phenylindole dihydrochloride (DAPI, Thermo Fisher Scientific, no. D1306) and anti−rabbit Alexa 488−conjugated antibody (Abcam, ab150157) were purchase for the immunofluorescence assay.

### 4.2. Cell Culture

The HL−60, K562, CA46, A549, SH−SY5Y, MCF−7, Siha, and MDA−MB−231 cell lines were obtained from the American Type Culture Collection (ATCC, Rockville, MD). The mitoxantrone−resistant acute promyelocytic leukemia cell line HL60/MX2 was a gift from the Shanghai Institute of Material Medica. The HL−60, HL60/MX2, K562, and CA46 cell lines were cultured in RPMI−1640 medium (Gibco, Carlsbad, CA) supplemented with 10% fetal bovine serum (Gibco, Carlsbad, CA), and other cell lines were grown in Dulbecco’s modified Eagle’s medium (D−MEM, Gibco Carlsbad, CA) supplemented with 10% fetal bovine serum. All cell lines were cultured with 5% CO_2_ at 37 °C.

### 4.3. Cloning, Expression, and Purification

The gene encoding the ATPase region of *h*Topo IIα (residues 29−428) was amplified by polymerase chain reaction using synthetic primers: F−*h*TopoIIα−29, 5′−GAGCAGCTAGCT CTGTGAGAGAGTGTATCAG−3′; R−*h*TopoIIA−428,5′−GTCGGCCTCGAGTGATGAACA CTTCTTATTC−3′. The resulting Nhe I/Xho I fragment was subcloned into Nhe I/Xho I sites of the pET28α−expression vector, yielding a fusion protein of 400 amino acids with N−terminal His−tags (6x His). Transformed *E. coli* BL21 (DE3) cells were cultured in Luria−Bertani medium containing 50 mg/L kanamycin and then induced with 0.3 mmol/L IPTG for 20 h (*h*Topo IIα−ATPase) or 1.0 mmol/L IPTG for 6 h at 30 °C. The collected cells were lysed, purified using a Ni^2+^−NTA agarose (GE company) column, and detected using SDS−PAGE.

GST−Hsp90 N (9−236) was a gift from William Sessa (Addgene plasmid no. 22481; http://n2t.net/addgene:22481, accessed on 29 April 2022; RRID: Addgene_22481). The fusion proteins were expressed and purified by glutathione affinity chromatography as described in the literature [57]. The GST tag of the fusion protein was cleaved by digestion with thrombin (T8021, Solarbio) at 20 ℃ for 12 h. The sample solution was isolated using a GSTrap FF column (17−5130−01, GE Healthcare). Hsp90α N−ATPase (9−236) protein was collected and concentrated using Millipore Amicon Ultra (0.5 mL, 3 kd, UFC501096−1) and stored in storage buffer (100 mM Tris, 20 mM KCl, 1 mM MgCl2, pH 7.4).

### 4.4. Structural Alignment of Topo IIα ATPase and Hsp90α N−ATPase

The structures of Topo IIα ATPase (PDB entry 1ZXM) and the Hsp90α N−ATPase (PDB entry 3T1K) were used to add hydrogen, assign bond orders, create zero−order bonds to metals, generate het states using epic pH = 7.0 ± 2.0, remove water, retain one of the main chains, and refine the structure using the Protein Preparation Wizard module; both proteins were aligned via the Align Bind Sites module in the Schrodinger software. We detected the ligand automatically and aligned residues within 5.0 Å from the ligand. A diagram of the alignment of proteins was generated using PyMOL.

### 4.5. Molecular Docking Analysis

The molecular docking analysis was performed using Glide of Schrodinger software. The Receptor Grid Generation panel was used to set up the grid generation job with the prepared structure, Topo IIα ATPase (PDB entry 1ZXM, https://www1.rcsb.org/structure/1ZXM, accessed on 29 April 2022, Released: 2005-08-23) and Hsp90α N−ATPase (PDB entry 3T1K, https://www1.rcsb.org/structure/3T1K, accessed on 29 April 2022. Released: 2012-01-25). The ANP was picked to define the enclosing box. QA was prepared using a LigPrep panel, possible states were generated at the target pH = 7.0 ± 2.0, and metal−binding states were added using Epic and OPLS3 force fields for QA. Molecular docking was carried out using the Ligand Docking panel. The ligand sampling was set to flexible and the precision was set to SP precision. All other parameters followed the default settings.

### 4.6. Colorimetric Determination of ATPase Activity

Inhibition of *h*Topo IIα ATPase, hHsp90α ATPase, and hHsp90α activity was detected using the malachite green phosphate assay [43]. ATPase activity was measured by the detection of free inorganic phosphate (Pi), which was released during the reaction. In brief, Topo IIα ATPase or Hsp90α was incubated with 100 μM compounds in assay buffer (30 mM Tris−HCl pH 7.8, 150 mM KCl, 10 mM MgCl2, 0.5 mM DTT, and 0.25 mg/mL BSA) containing 1 mM ATP (Sigma−Aldrich, A7699, inorganic phosphorus ≤ 0.1%). After incubation at 37 ℃ for 45 min (Topo IIα ATPase) or 3 h (Hsp90α N−ATPase and Hsp90α), malachite green reagent (malachite green (0.0812%, *w*/*v*), polyvinyl alcohol (2.32%, *w*/*v*), ammonium molybdate (5.72%, *w*/*v*, in 6 M HCl), and ddH_2_O mixed at a ratio of 2:1:1:2 was freshly prepared) was added, followed by the injection of 34% sodium citrate. The mixture was then incubated for 15 min at room temperature, and the absorbance at 620 nm was measured on a PowerWave XS2 (BioTek). All experiments were performed in parallel triplicate. Moreover, 200 μM 1,4−naphthoquinone (1,4−NQ) and 100 μM 17−AAG were used as positive controls for the Topo II and Hsp90 inhibitor screening, respectively. To facilitate the evaluation of the activity of a large number of compounds, the final inhibitory activity of the compound was evaluated with a relative inhibition rate (RIR). RIR compd% = IRcompd/IRpositive ×100%.

### 4.7. Cell Proliferation Assay

The cell proliferation assay was evaluated using the MTT assay as described by Mosmann, with modifications [58]. Cells were seeded into 96−well plates (5 × 103 cells/well) and exposed to various concentrations of compounds. After 48 h of treatment, 20 μL of 2.5 mg/mL MTT reagent was added to each well, and the cells were further incubated for 4 h at 37 ℃. The cells in each well were then treated with 100 μL of dimethyl sulfoxide (DMSO), and the optical density (OD) was recorded at 570 nm using a microplate reader (Bio−Tek). All experiments were performed in parallel three times, and the IC_50_ values were derived from the mean OD values of triplicate tests versus the drug concentration curves.

### 4.8. Topo II−Mediated DNA Relaxation Assay

An *h*Topo IIα relaxation assay kit was used to determine the effects of the compounds. The relaxation assay was performed according to the manufacturer’s instructions with minor modifications. The assay was performed in a final volume of 20 μL in Topo II reaction buffer (5 mM Tris−HCl, pH 7.5, 12.5 mM NaCl, 1 mM MgCl_2_, 2 mM ATP, 0.5 mM dithiothreitol, and 10 μg/mL BSA) with 0.2 μg pBR322 DNA. Compounds were included in the reactions at a constant solvent volume. The reaction was initiated by the addition of 1 unit of human Topo IIα and incubated for 30 min at 37 ℃. The reaction was terminated with a stop buffer (5% sarkosyl, 0.0025% bromophenol blue, and 25% glycerol). Reaction products were analyzed with a 1% agarose gel in TAE buffer (40 mM Tris−acetate, 2 mM EDTA). Gels were stained for 30 min in TAE buffer with Gel Red. DNA bands were visualized through transillumination with UV light and then photographed using a Tanon−4200SF gel imaging system (Tanon, Shanghai, China).

### 4.9. Topo II−Mediated DNA Cleavage Assay

A cleavage assay was performed according to the manufacturer’s instructions with minor modifications similar to the relaxation assay. In contrast, the reaction was initiated by the addition of 10 units of human Topo IIα and incubated for 6 min at 37 ℃. Then 2 μL 10% SDS, 2 μL 250 mM NaEDTA (pH 8.0), and 2 μL protease K (1 mg/mL) were added to the volume in sequence and incubated for 30 min at 45 ℃. The reactions were stopped, processed, and subjected to gel electrophoresis. The gels were stained for 30 min in TAE buffer with Gel Red. DNA bands were visualized through transillumination with UV light and then photographed using a Tanon−4200SF gel imaging system (Tanon, Shanghai, China).

### 4.10. DNA−Unwinding Assay

The DNA−unwinding assay was performed according to the manufacturer’s instructions with minor modifications. The assay was performed using eukaryotic DNA Topo I to clarify the intercalation properties of the compounds. In the presence of the DNA intercalators, relaxed plasmid DNA appears to be supercoiled plasmid DNA by treatment with Topo I. Reactions were carried out in the presence of ethidium bromide. All reaction mixtures contained 1% DMSO (final concentration). Following a 5 min incubation of DNA with drugs or DMSO, topoisomerase I was added, and the reactions were incubated for up to 30 min at 37 ℃. The reactions were stopped, processed, and subjected to gel electrophoresis. DNA bands were visualized through transillumination with UV light and then photographed using a Tanon−4200SF gel imaging system (Tanon, Shanghai, China).

### 4.11. Western Blotting

Cells treated with compounds or mock were collected and lysed in the RIPA lysis buffer (Bioteke, China), and the protein concentrations were determined using a BCA protein assay kit (Pierce, USA). In total, 40 μg of protein were resolved by 10% SDS−PAGE and transferred to 0.22−μm immobilon polyvinyl difluoride (PVDF) membranes. The blots were blocked with 5% BSA for 1 h at 25 °C and probed with primary antibodies (1:1000) at 4 °C overnight. After three washes, the blots were subsequently incubated with the corresponding secondary antibodies (1:3000) for 1 h at 25 ℃. The protein bands were visualized using a chemiluminescence substrate, and images were acquired using a Tanon−4200SF gel imaging system (Tanon, Shanghai, China).

### 4.12. Immunofluorescence

Cells grown on glass coverslips were fixed in 4% paraformaldehyde/PBS for 15 min, permeabilized with 0.1% Triton−X100/PBS at 37 °C for 30 min, and finally blocked with 5% goat serum/PBS at 37 °C for 2 h. Immunofluorescence was performed using standard methods, and the slides were incubated alternately with phospho−γH_2_AX(Ser139) at 37 °C for 3 h. The glass coverslips were washed six times with blocking buffer and then incubated with anti−rabbit Alexa 488−conjugated antibody and 2 μg/mL DAPI at 37 ℃ for 3 h. The glass coverslips were again washed six times with a blocking buffer. Digital images were recorded using an LSM710 microscope (Zeiss, Germany) and analyzed with ZEN software. Fifty nuclei were counted in each group, and the s.e.m. was calculated from three replicates. Frequency distribution graphs were plotted using GraphPad Prism 7 (GraphPad Software Inc., San Diego, CA, USA).

### 4.13. Wound−Healing Assay

A549 cells were cultured in 35−mm tissue−culture dishes to 90% confluence. A sterile 200 µL microtip was used to make a wound through the monolayer by scratching cells at an angle of 30°. The scratched cells were removed by rinsing with a fresh medium. The cells were treated with different concentrations of QA in a fresh medium and allowed to proliferate. Photography was performed at 40× magnification with an inverted microscope (Nikon, Japan) to assess the healing of the wound at time intervals of 0, 12, 24, and 48 h. Data presented herein show only the 24 and 48 h treatments with different concentrations of QA and the best of three independent experiments.

### 4.14. Competitive Displacement Assays Using Geldanamycin−FITC

FP (fluorescence polarization) assays were performed under the following conditions: the assay buffer (HFB) contained 20 mM HEPES, pH 7.3, 50 mM KCl, 5 mM MgCl_2_, 20 mM Na_2_MoO_4_, and 0.01% NP−40. Before each use, 0.1 mg/mL BSA (bovine serum albumin) and 2 mM DTT (dithiothreitol) were freshly added, and each 384−well microplate (black, round bottom, nonbinding surface, Corning no. 4514) contained 40 nM GM−FITC (geldanamycin−FITC), 80 nM Hsp90α N−ATPase protein, and the tested compounds in a final volume of 20 µL. Background wells (buffer only), tracer controls (free, GM−FITC only), bound wells (GM−FITC and Hsp90α N−ATPase), and test wells (GM−FITC, compound, and Hsp90α N−ATPase) were set. The plate was mixed on a shaker for 3 h. Polarization was measured on a Victor^TM^ X5 multilabel plate reader instrument (Perkin Elmer) at room temperature with an excitation wavelength of 485 nm and an emission wavelength of 535 nm. The assays were performed in parallel three times. The displacement activity% = [1 − (FP_test_ − FP_free_)/(FP_bound_ − FP_free_)] × 100.

### 4.15. Flow Cytometric Analysis

Cells were treated with 0.5, 1, 2, 4, and 8 μM QA for 48 h and collected. For the apoptosis experiments, cells were washed in PBS, added to 0.5 mL binding buffer, and resuspended in a staining solution (Annexin V−FITC and PI) for 15 min at room temperature in the dark. Cells were analyzed by flow cytometry using an EPICS XL flow cytometer (Beckman Coulter, Brea, CA, USA).

### 4.16. Statistical Analysis

Data are presented as the mean values ± standard deviation. A two−tailed Student’s *t*-test was used for comparison of treatment versus control groups. These analyses were performed using GraphPad Software 7 (GraphPad Inc. China). Differences were considered significant at *p* < 0.05.

## Figures and Tables

**Table 1:** Information on active compounds in the preliminary screening of the kinase library and FDA drug library.

Location	Name	CAS	Kinase	Topo II ATPase RIR (%) *	HL60 IC_50_ (μM) **
K−B6	Staurosporine	62996−74−1	Pan−specific	111.2	<0.01
K−C2	Tyrphostin 47	122520−86−9	EGFRK	90.3	>25
K−C3	Tyrphostin 51	122520−90−5	EGFRK	85.8	>25
K−C10	PKC−412	120685−11−2	PKC	68.4	0.75
K−C11	Piceatannol	10083−24−6	Syk	70.5	12.07
K−Dl	AG−490	133550−35−3	JAK−2	61.6	18.97
K−D6	Wortmannin	19545−26−7	PI 3−K	66.8	13.32
K−D7	GF 109203X	133052−90−1	PKC	64.7	9.51
K−D8	Hypericin	548−04−9	PKC	75.8	>25
K−D9	Ro 31−8220	138489−18−6	PKC	99.7	1.18
K−D10	Sphingosine	123−78−4	PKC	94.3	11.10
K−Dl 1	H−89	127243−85−0	PKA	77.1	13.24
K−D12	H−8	84478−11−5	PKA, PKG	62.3	>25
K−F9	Palmitoyl−d,L−carnitine	6865−14−1	PKC	109.4	>25
K−F12	Daidzein	486−66−8	CK2	101.2	3.02
2−B8	Fluphenazine 2HC1	146−56−5		64.1%	13.38
2−G7	Atovaquone	95233−18−4		47.2%	9.43
2−H10	Bopindolol malonate	62658−64−4		41.2%	13.23
2−H11	Guanfacine HCl	29520−14−7		64.5%	>25
3−C11	Gallamine triethiodide	65−29−2		56.2%	>25
3−D11	Flunarizine−2HCl	30484−77−6		42.4%	22.73
3−E6	Trifluoperazine 2HCl	440−17−5		58.1%	>25
3−H6	Actarit	18699−02−0		45.4%	8.02
4−C3	Raloxifene HCl	82640−04−8		47.6%	7.20
5−E4	4−aminosalicylic acid	65−49−6		40.2%	>25
5−E5	5−aminosalicylic acid	89−57−6		42.3%	>25
5−E6	Ampicillin trihydrate	7177−48−2		42.5%	>25
5−E11	Atracurium besylate	64228−81−5		88.9%	22.86
6−B10	Doxazosin mesylate	77883−43−3		47.6%	19.73
7−F2	Quinacrine 2HCl 2H_2_O	83−89−6		42.3%	5.22
7−H3	Streptomycin sulfate	3810−74−0		71.6%	>25
7−H4	Sulfadoxine	2447−57−6		71.2%	>25
7−H5	Sulfadiazine	68−35−9		71.0%	>25
7−H6	Sulfadimethoxine	122−11−2		73.9%	>25
7−H7	Sulfasalazine	599−79−1		70.8%	>25
7−H8	Tamsulosin HCl	106463−17−6		71.0%	>25
7−H9	Telmisartan	144701−48−4		79.4%	>25
7−H10	Tenoxicam	59804−37−4		76.1%	>25
7−H11	Terazosin HCl	63590−64−7		70.6%	>25
8−H9	L−thyroxine	51−48−9		42.0%	>25
VP16					0.17

* The malachite green phosphate assay of Topo II ATPase: 200 μM 1,4−naphthoquinone (1,4−NQ) was used as a positive control, and the relative inhibition rate (RIR) was calculated. RIR compd% = (IRcompd/IRpositive) × 100%. ** The MTT assay for HL−60 (48 h).

## Data Availability

Data are contained within the article or Supplementary Materials. The samples of the compounds used and/or analyzed during the current study are available from the corresponding author upon reasonable request.

## Supplementary Materials

The following supporting information can be downloaded at: https://www.mdpi.com/article/10.3390/molecules27175561/s1, Figure S1: Structural alignment of Topo IIα ATPase and Hsp90 N−ATPase; Figure S2: Results of preliminary screening of Topo IIα inhibitors in the existing drug library by the malachite green phosphate assay; Figure S3: Chemical structure of kinase inhibitors with inhibition on Topo II ATPase; Figure S4: Structures of drugs with inhibitory effects on Topo II ATPase. Figures S5: Original full−length gels of the figure (in white boxes) in the article. S6: Original full−length gels of the figure (in white boxes) in the article. S7: Original full−length gels of the figure (in white boxes) in the article. S8: Original full−length gels of the figure (in white boxes) in the article. S9: Original full−length gels of the figure (in white boxes) in the article. S10: Original full−length gels of the figure (in white boxes) in the article. S11: Original full−length gels of the figure (in white boxes) in the article. S12: Original full−length gels of the figure (in white boxes) in the article.
