# Supplementary material for: Discovery of Quinacrine as a Potent Topo II and Hsp90 Dual-Target Inhibitor, Repurposing for Cancer Therapy
Source: Molecules. 2022 Aug 29;27(17):5561. doi: 10.3390/molecules27175561 (PMC9458065; doi:10.3390/molecules27175561)
Supplement: Supplementary file 1 [file molecules-27-05561-s001.zip › molecules-1865913-supplementary.pdf]

molecules

MDPI

Supplementary Materials

Discovery of Quinacrine as a Potent Topo II and Hsp90 Dual-target Inhibitor Repurposing for Cancer Therapy

Xin Pan [a], Teng-yu Mao [a], Yan-wen Mai [a], Cheng-cheng Liang [a], Wei-hao Huang [a], Yong Rao [a], Zhi-shu Huang[a] and Shi-liang Huang [a,b*]

[a] School of Pharmaceutical Sciences, Sun Yat-sen University, Guangzhou 510006, People's Republic of China
[b] Guangdong Provincial Key Laboratory of New Drug Design and Evaluation, Guangzhou 510120, China.
* Correspondence: lsshsl@mail.sysu.edu.cn. Tel.: 8620- 39943053, Fax.: 8620-39943056, e-mail:

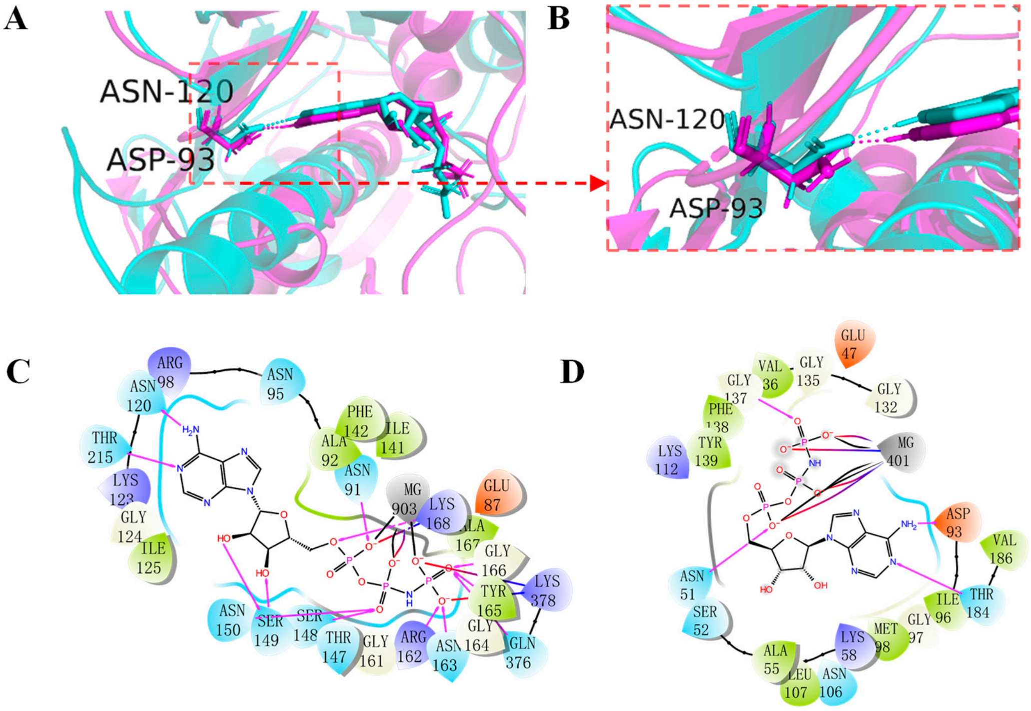

Figure S1. Structural alignment of Topo IIα ATPase and Hsp90 N-ATPase. (A-B) Structural alignment of the ATPase domains of Topo IIα (purple) and Hsp90α (blue). The hydrogen bonds between QA and ASN120 (Topo IIα ATPase) and ASP93 (Hsp90 N-ATPase) are indicated by dotted lines. (C) Binding mode of ANP with Topo II (PDB: 1ZXM). (D) Binding mode of ANP with Hsp90 (PDB: 3T1K).

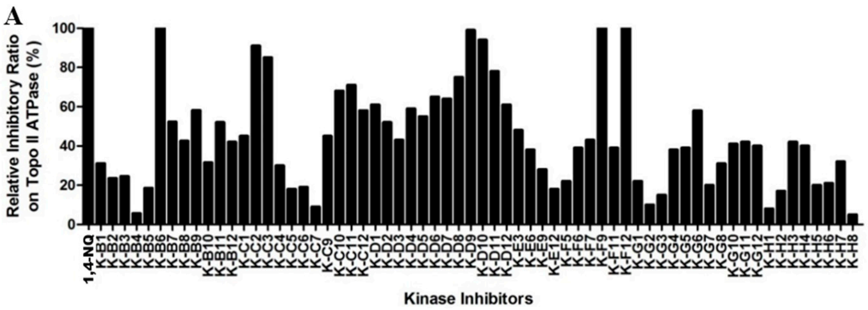

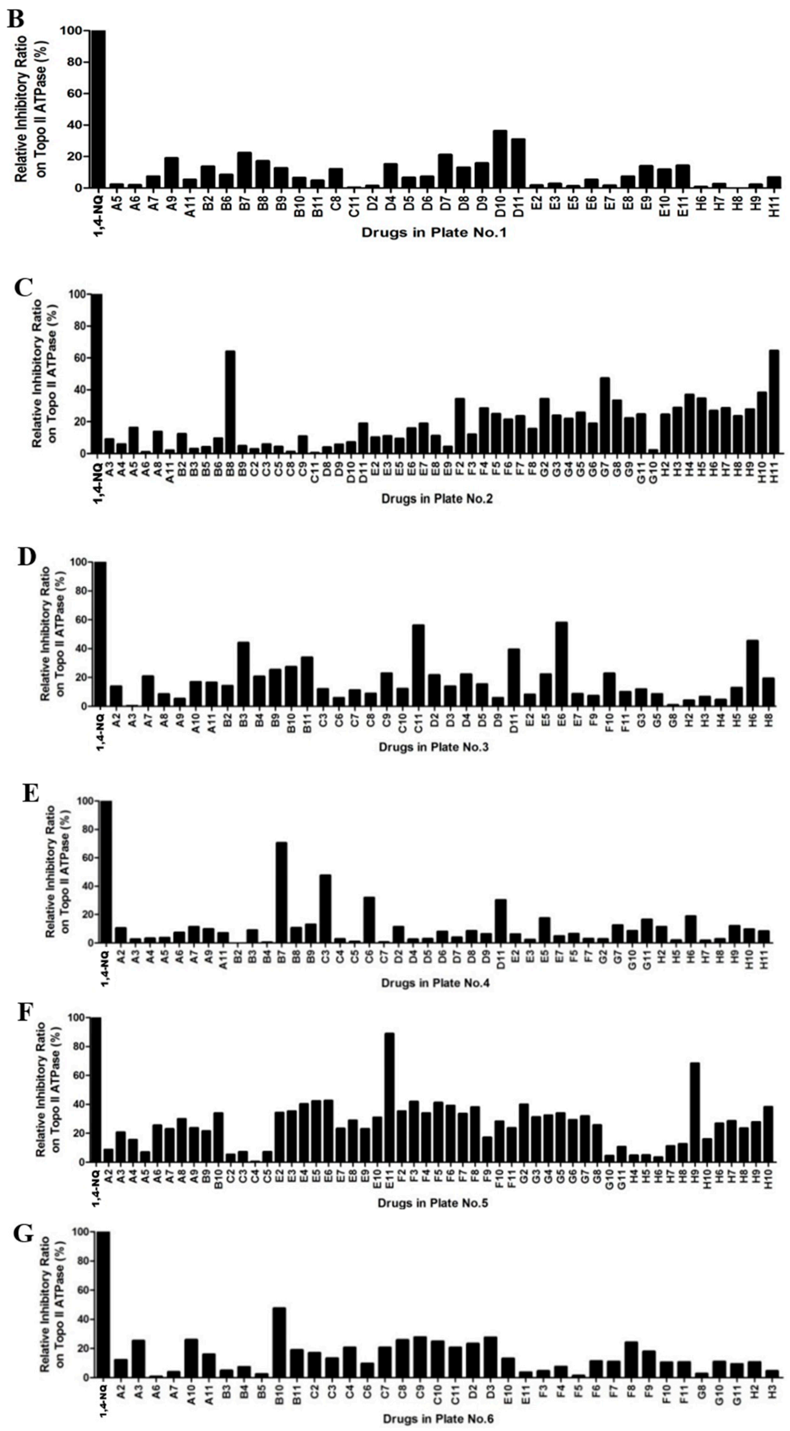
B
Relative Inhibitory Ratio on Topo II ATPase (%)
Drugs in Plate No.1
C
Relative Inhibitory Ratio on Topo II ATPase (%)
Drugs in Plate No.2
D
Relative Inhibitory Ratio on Topo II ATPase (%)
Drugs in Plate No.3
E
Relative Inhibitory Ratio on Topo II ATPase (%)
Drugs in Plate No.4
F
Relative Inhibitory Ratio on Topo II ATPase (%)
Drugs in Plate No.5
G
Relative Inhibitory Ratio on Topo II ATPase (%)
Drugs in Plate No.6

H

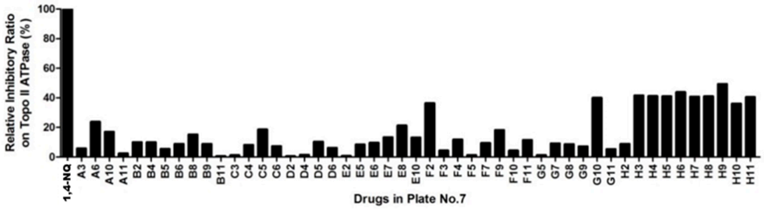

I

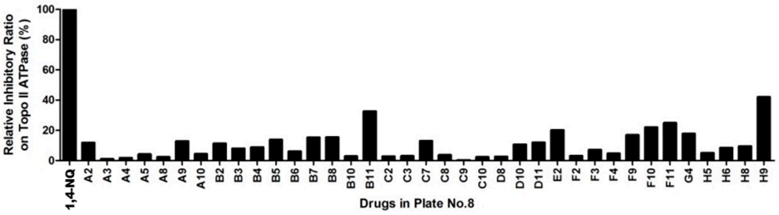

Figure S2. Results of preliminary screening of Topo IIα inhibitors in the existing drug library by the malachite green phosphate assay (Due to space limitations, the results for compounds with an inhibition rate below 0 are not shown in the Figure). (A) Relative inhibitory ratio of kinase inhibitors on ATPase. (B-I) Inhibitory effect of FDA-approved drugs on Topo II ATPase activity. Topo II ATPase activity at 100 μM of compounds and 200 μM of 1,4-NQ as positive control were tested by the malachite green phosphate assay. After 45 min, the reaction was stopped and the OD620 value measured(A-I).

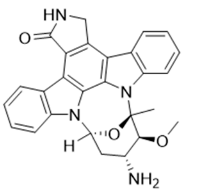

K-B6
Staurosporine

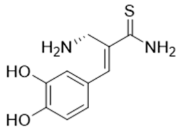

K-C2
Tyrphostin 47

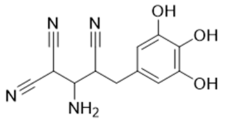

K-C3
Tyrphostin 51

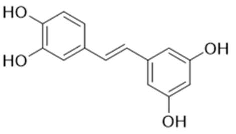

K-C11
Piceatannol

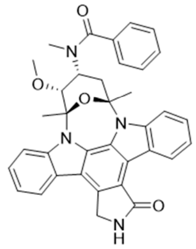

K-C10
PKC-412

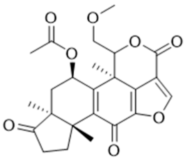

K-D6
Wortmannin

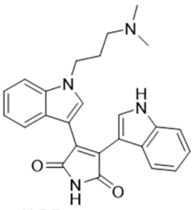

K-D7
GF 109203X

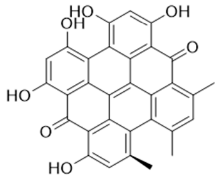

K-D8
Hypericin

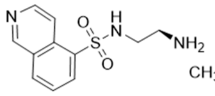

K-D12
H-8

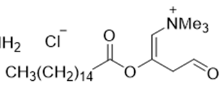

K-F7
Palmitoyl-DL-carnitine

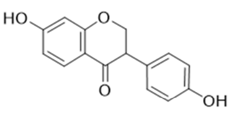

K-F12
Daidzein

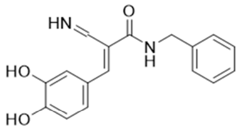

K-D1
AG490

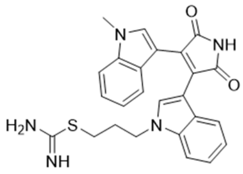

K-D9
Ro 31-8220

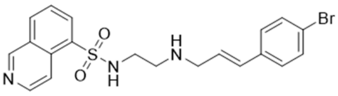

K-D11
H-89

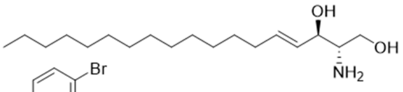

K-D10
Sphingosine

Figure S3. Chemical structure of kinase inhibitors with inhibition on Topo II ATPase.

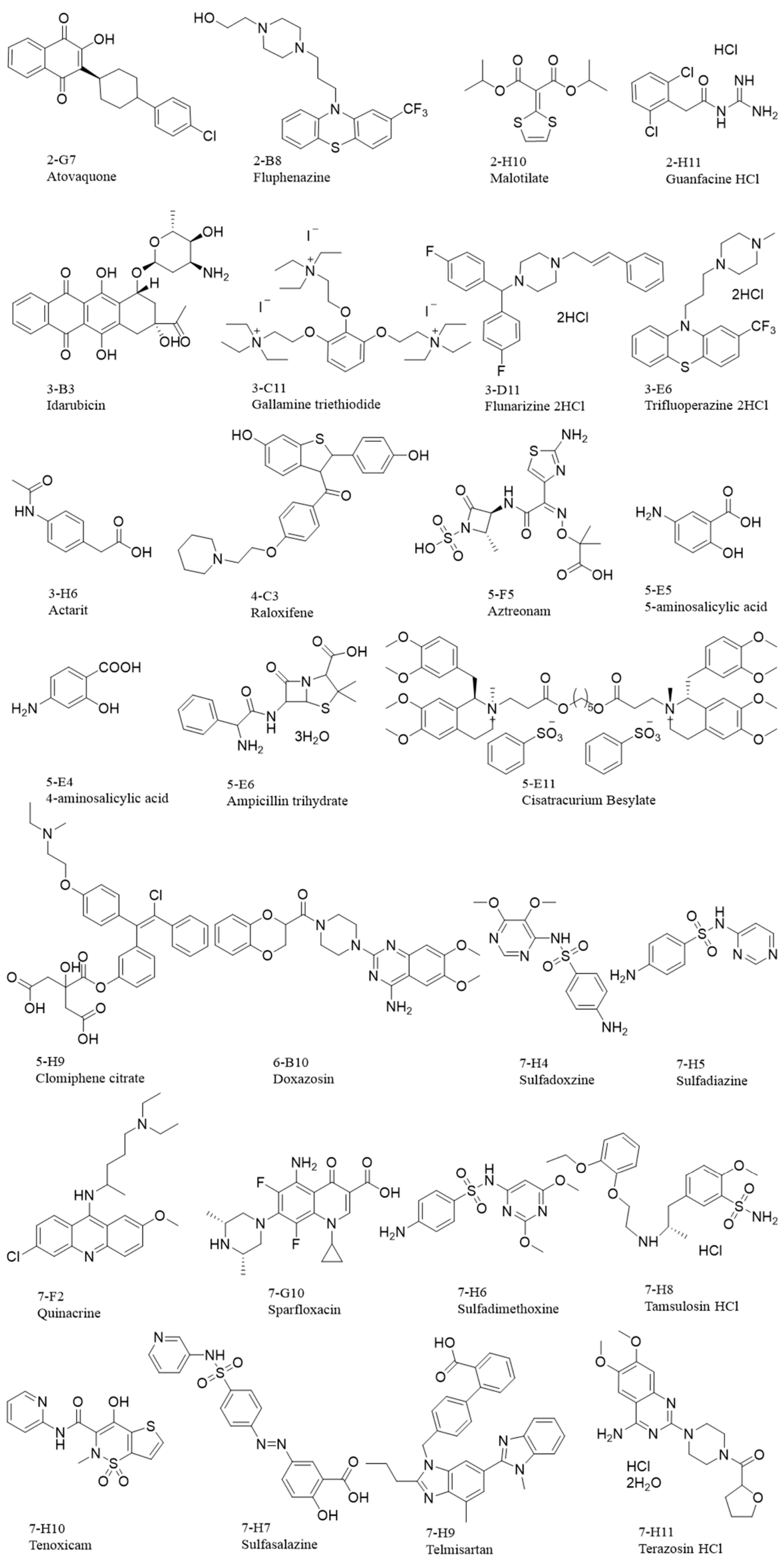

Figure S4. Structures of drugs with inhibitory effect on Topo II ATPase.

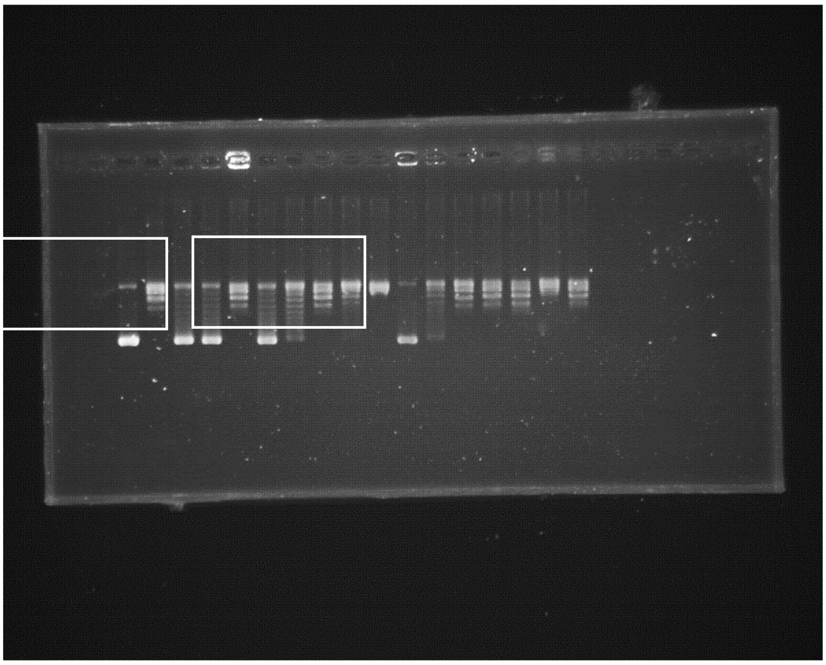

Figure S5. Original full-length gels of Figure 1A (in white boxes) in the article.

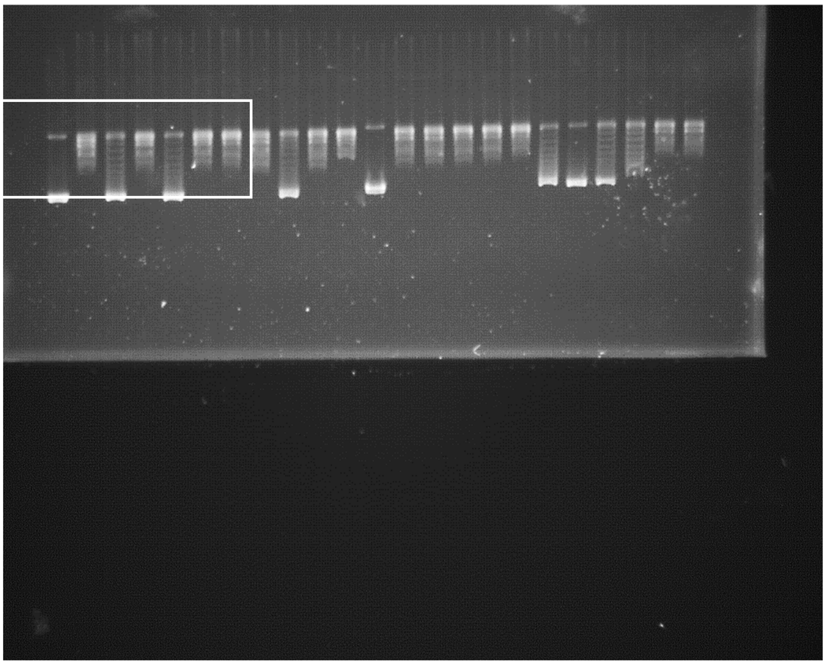

Figure S6. Original full-length gels of Figure 1B (in white boxes) in the article.

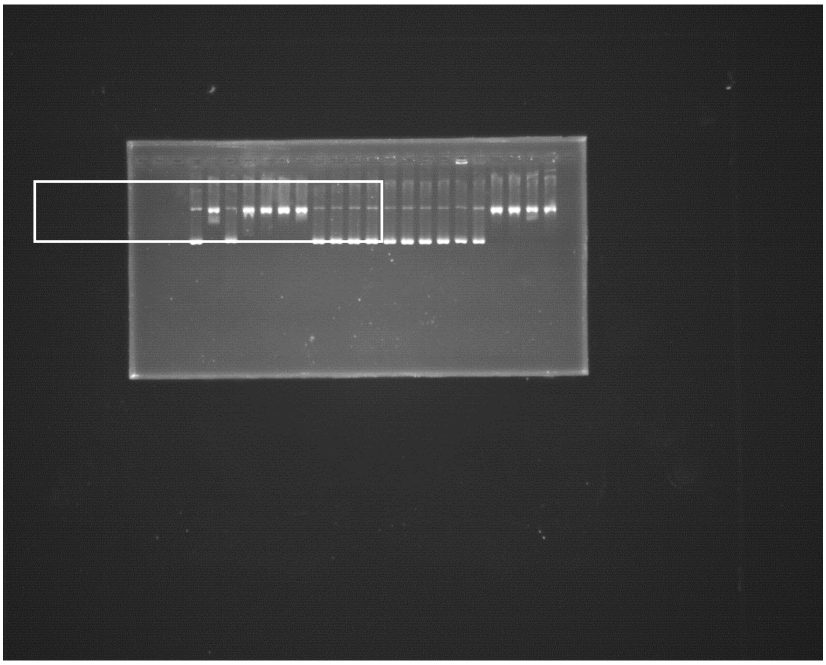

Figure S7. Original full-length gels of Figure1C (in white boxes) in the article.

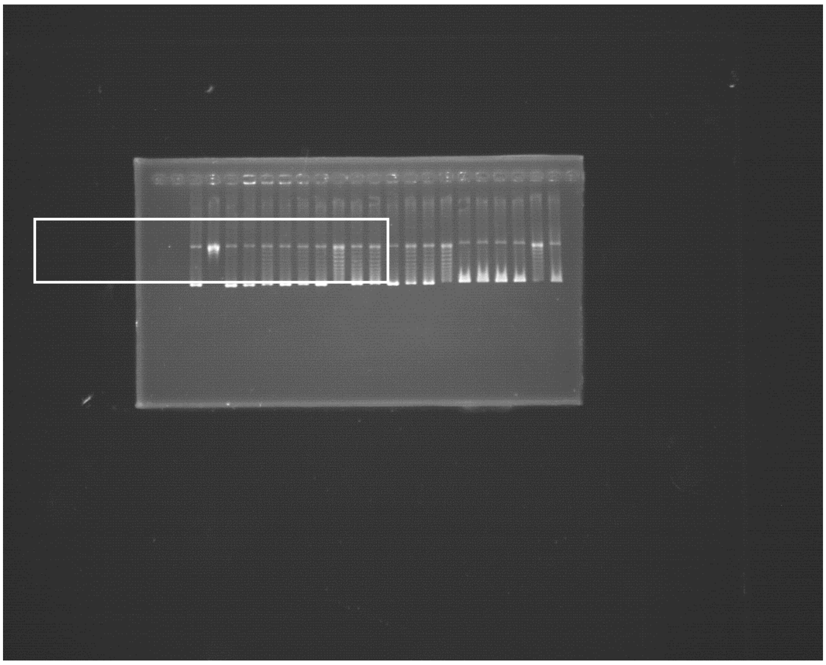

Figure S8. Original full-length gels of Figure1D (in white boxes) in the article.

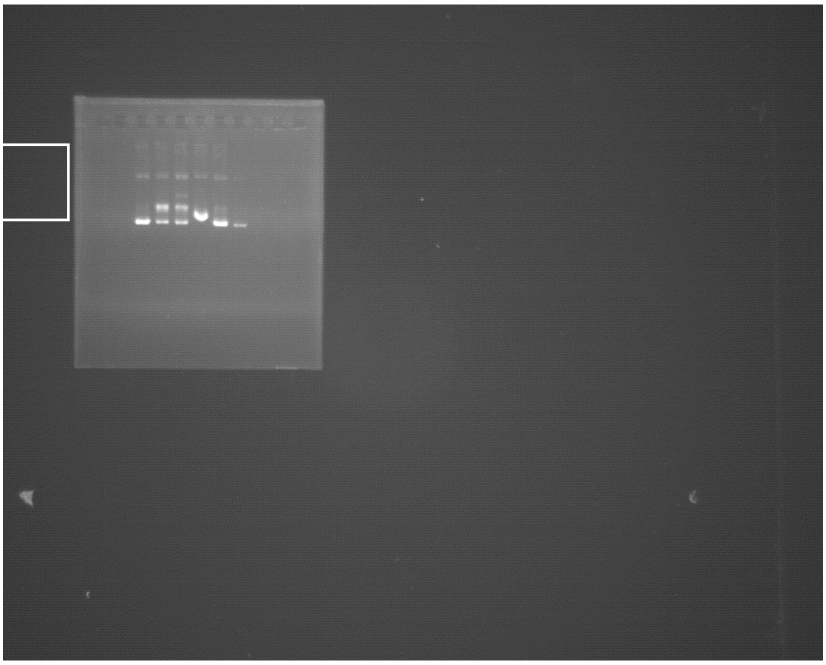

Figure S9. Original full-length gels of Figure2A (in white boxes) in the article.

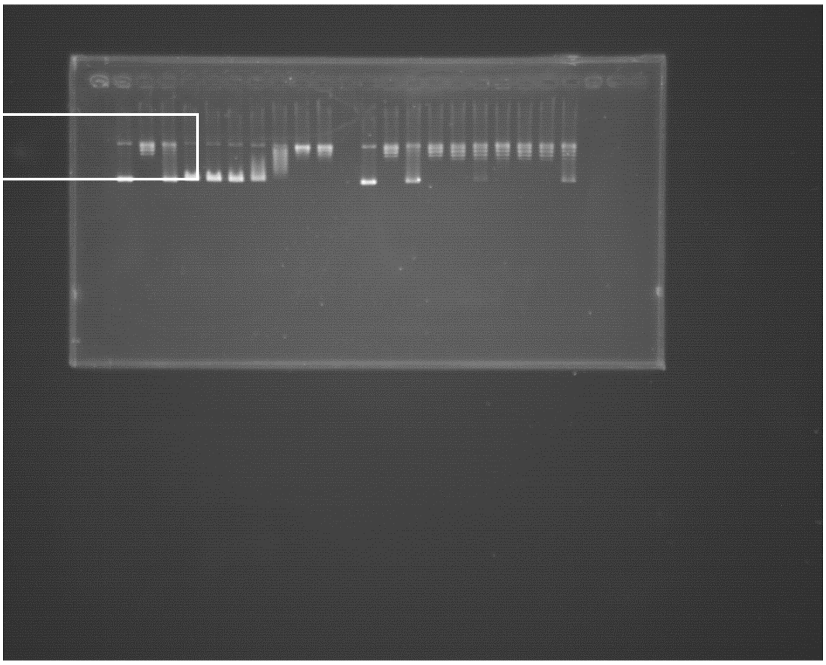

Figure S10. Original full-length gels of Figue 2B (in white boxes) in the article.

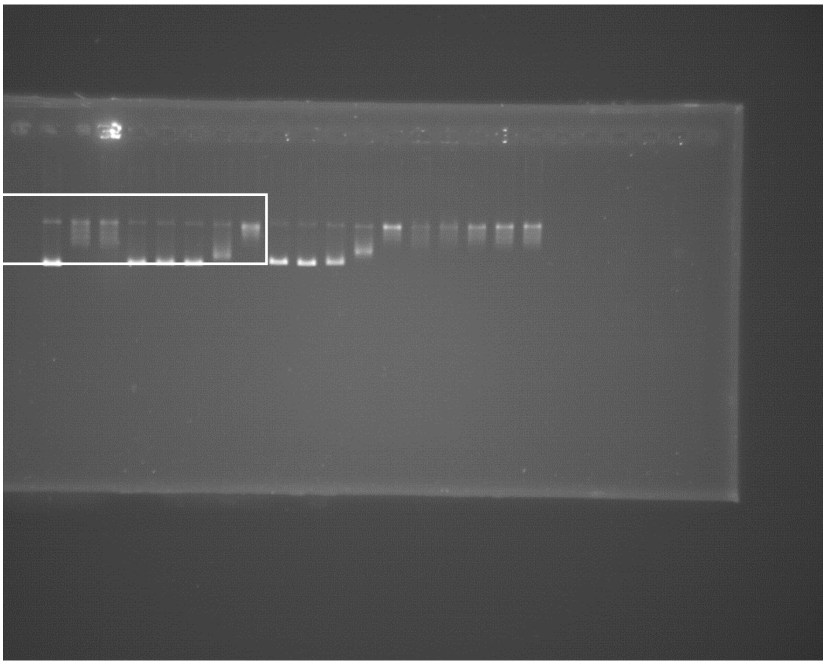

Figure S11. Original full-length gels of Figue 2C (in white boxes) in the article.

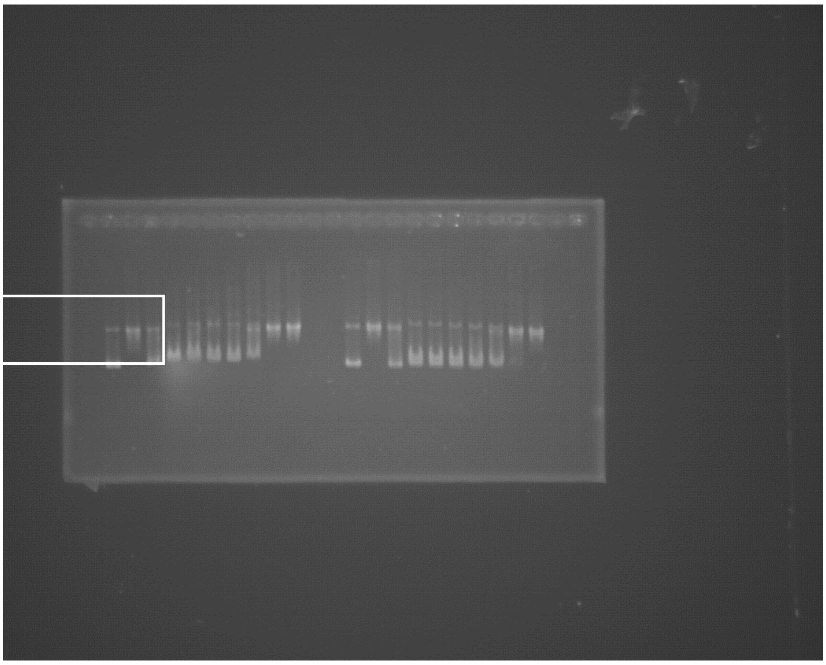

Figure S12. Original full-length gels of Figure2D (in white boxes) in the article.
